# Synergistic Effects of Joint-Biased Rehabilitation and Combined Transcranial Direct Current Stimulation (tDCS) in Chronic Ankle Instability: A Single-Blind, Three-Armed Randomized Controlled Trial

**DOI:** 10.3390/brainsci15050458

**Published:** 2025-04-27

**Authors:** Yunseo Kim, Hyunjoong Kim, Jihye Jung, Seungwon Lee

**Affiliations:** 1 Department of Physical Therapy, Graduate School of Sahmyook University, 815, Hwarang-ro, Seoul 01795, Republic of Korea; kimys000302@gmail.com; 2 Department of Senior Exercise Prescription, Gwangju Health University, 73, Bungmun-daero 419, Gwangju 62287, Republic of Korea; hjkim@ghu.ac.kr; 3 Institute of SMART Rehabilitation, Sahmyook University, 815, Hwarang-ro, Seoul 01795, Republic of Korea; jihye3752@gmail.com; 4 Department of Physical Therapy, College of Future Convergence, Sahmyook University, 815, Hwarang-ro, Seoul 01795, Republic of Korea

**Keywords:** ankle joint, joint instability, transcranial direct current stimulation, balance

## Abstract

**Background/Objectives:** The ankle joint is among the most frequently injured joints in daily life, with approximately 25% of young adults reporting chronic ankle instability (CAI). This study investigated the effects of transcranial direct current stimulation (tDCS), a type of non-invasive brain stimulation (NIBS) technique, combined with joint mobilization and active joint mobilization on CAI. **Methods:** A total of 36 participants (mean age: 20.81 years; 63.89% female; mean body mass index: 21.68) were randomly divided into three groups: (1) tDCS with joint mobilization (*n* = 12); (2) active joint mobilization (*n* = 12); and (3) tDCS with active joint mobilization (*n* = 12). Dynamic balance, range of motion (ROM), static balance, and ankle instability (Cumberland Ankle Instability Tool, CAIT) were evaluated at multiple time points. Interventions were conducted three times per week, for 15 min per session, over four weeks (12 sessions total). **Results:** All three groups showed significant improvements over time in dynamic balance, ankle instability, ROM, and static balance (*p* < 0.05). However, no significant interaction effects were observed between time and group (*p* > 0.05). The tDCS with active joint mobilization group demonstrated the largest effect sizes across most outcome measures, particularly for ankle instability, ROM, and static balance, in both immediate and post-intervention assessments. **Conclusions:** tDCS combined with active joint mobilization appears to be particularly effective in improving CAI. This approach, targeting both top-down mechanisms through non-invasive brain stimulation and local joint function, offers a promising alternative to traditional interventions that focus solely on the ankle joint. This study was registered with the Clinical Research Information Service (CRIS) under the identifier KCT0009566.

## 1. Introduction

The ankle joint is highly susceptible to injury because of frequent jump-landing activities and sudden changes in direction, which impose significant biomechanical stress [1]. Chronic ankle instability (CAI) affects approximately 25% of young adults aged 18–24 years, and up to 36% of individuals with a history of ankle sprain experience symptoms of CAI [2]. Among the contributing factors, participation in recreational physical activities and sports has been identified as a significant risk factor for lateral ankle sprains, which may lead to CAI [3]. The hallmark characteristics include neuromuscular control deficits, peri-ankle muscle weakness [4], restricted dorsiflexion range of motion [5], and proprioceptive impairments affecting postural control [6]. Ankle sprains commonly damage the lateral ligaments, particularly the anterior talofibular ligament, leading to mechanical instability and altered proprioceptive input. These structural changes contribute to neuromuscular deficits and impaired postural control, thus forming the underlying pathophysiological basis of CAI [6].

CAI is commonly classified as mechanical ankle instability (MAI), defined as ligamentous laxity and altered joint mechanics, or functional ankle instability (FAI), which refers to perceived instability without mechanical deficits [7,8]. These subtypes often coexist and are associated with impaired proprioception and neuromuscular control, which may further compromise balance and postural regulation. Among these functional impairments, the characteristic “giving-way” episodes observed in individuals with FAI are primarily attributed to deficits in neuromuscular control, including impairments in proprioception, muscle strength, and postural control [9]. Electrophysiological investigations have demonstrated diminished cortical excitability in motor cortical regions associated with ankle-stabilizing muscles among patients with CAI [10,11].

Non-invasive brain stimulation (NIBS), particularly transcranial direct current stimulation (tDCS), is a direct modality for modulating motor cortical excitability [12]. Despite stimulation limitations, tDCS has the advantages of cost-effectiveness, portability, and bidirectional modulatory capabilities [13,14], with demonstrated efficacy in motor function enhancement [15,16]. Although tDCS is widely regarded as a safe and non-invasive technique, recent studies have reported mild and transient adverse effects, including itching, tingling, and burning sensations at the stimulation site, as well as occasional dizziness and blurred vision [17].

Volitional movement has been identified as a more effective approach than passive movement for enhancing the excitability of the primary motor cortex [18]. Research has demonstrated superior outcomes in patients with CAI receiving active joint mobilization (AJM) with volitional movement compared to conventional joint mobilization, suggesting that heightened motor cortical activation facilitates neural circuit reorganization [19,20].

Although tDCS alone can modulate cortical excitability, its combination with task-specific physical training has been shown to synergistically enhance motor cortex excitability, improve motor learning, and strengthen sensorimotor integration, leading to substantial and long-lasting functional improvements [21]. In contrast to these safety-focused studies, previous applications of tDCS in joint-related rehabilitation have primarily targeted pain relief, such as post-stroke shoulder pain and chronic low back pain. However, this study shifts the focus toward enhancing motor cortical excitability through top-down neuromodulation to improve neuromuscular control in individuals with chronic ankle instability [22,23].

This study aimed to address the limitations of conventional CAI interventions by pursuing two objectives: (1) to compare the cortical activation effects of top-down modulation induced by tDCS and volitional movement, and (2) to evaluate the synergistic effects of combining tDCS with volitional joint mobilization. Using this approach, we sought to identify novel neuromodulatory strategies for improving neuromuscular control in individuals with FAI.

## 2. Materials and Methods

### 2.1. Study Design

**Figure 1:**
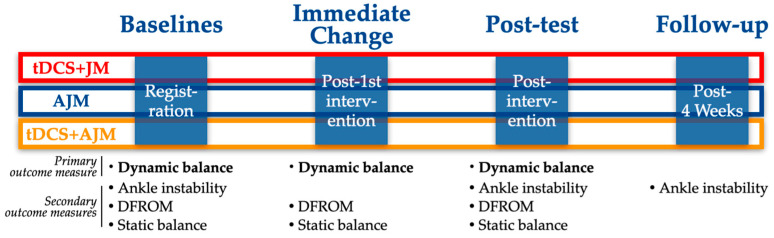
Schematic of the experimental design. AJM: active joint mobilization; DFROM: dorsiflexion range of motion; JM: joint mobilization; tDCS: transcranial direct current stimulation.

This investigation was a three-arm, single-blind, randomized controlled trial with a longitudinal, prospective assessment. The trial protocol was registered with the Clinical Research Information Service (CRIS), a primary registry of the World Health Organization International Clinical Trials Registry Platform (WHO ICTRP), on 24 June 2024 (KCT0009566), with initial participant enrollment commencing on 1 July 2024. Figure 1 provides a comprehensive overview of the experimental design and procedural timeline.

### 2.2. Participants and Ethics

The study population consisted of young adults aged 20–29 who had experienced ankle instability, recruited from Gwangju Health University located in Gwangju Metropolitan City, Republic of Korea. Participants were recruited voluntarily through advertisement posting disseminated throughout the university campus. Eligibility was assessed based on predetermined inclusion and exclusion criteria. All participants were screened for eligibility before enrollment in the experimental protocol [24,25,26].

In addition to the registered exclusion criteria, participants were screened for current use of medications that could influence neuromuscular function and for involvement in any concurrent physical therapy or rehabilitation program. Individuals who met any of these criteria were excluded from the eligibility screening process to reduce potential confounding factors.

#### 2.2.1. Inclusion Criteria

- Cumberland Ankle Instability Tool (CAIT) score of 24 or below
- A history of at least two ankle sprains on the same side within 2 years
- No other musculoskeletal injuries affecting the lower extremities

#### 2.2.2. Exclusion Criteria

- Ankle sprain occurring less than 6 months prior to study participation
- Sensory impairment or vestibular disorders
- History of surgery involving the back, hip, or knee
- Diagnosed neurological or psychiatric disorders
- Presence of metal implants in areas where electrical stimulation is applied

#### 2.2.3. Ethical Considerations

Before commencing the investigation, comprehensive ethical protocols were implemented to ensure the participants’ autonomy and protection. The principal investigator provided a thorough verbal explanation of the study’s objectives, methodological framework, anticipated outcomes, and procedural elements. Additionally, participants received detailed documentation outlining potential adverse events, discomfort, and corresponding risk-mitigation strategies.

Informed consent documentation was meticulously crafted using accessible terminology to facilitate complete comprehension by all participants. This process emphasized participants’ rights to data confidentiality, personal anonymity, and unrestricted access to consultations with the investigators. The participants were explicitly informed of their right to discontinue participation without prejudice or consequences at any juncture of the investigation.

The research protocol adhered rigorously to the ethical principles outlined in the Declaration of Helsinki. The study received formal ethical approval from the Institutional Review Board of Sahmyook University (approval number: SYU 2024-05-010-001, date: 27 May 2024), ensuring compliance with established standards for human subject research.

### 2.3. Sample Size

The required sample size was determined based on previous investigations examining tDCS interventions for individuals with ankle instability [26]. Given the limited availability of three-group design with repeated measurements, the sample size estimation was based on a two-group study and adjusted conservatively. Specifically, the effect size F = 0.3 was selected, which is more appropriate for repeated-measures ANOVA involving a between-within interaction. This conservative estimate was derived from analogous research and was chosen to avoid overestimating the intervention effect. Statistical power was maintained at 0.85 across the three experimental conditions. Using G*Power 3.1 software (Hein-rich-Heine-Universität Düsseldorf, Düsseldorf, Germany), the analysis indicated a required sample size of 30 participants. To mitigate the effect of potential participant withdrawal, this figure was increased by 20%, resulting in a final recruitment target of 36 individuals.

### 2.4. Randomization and Blinding

Participants who met the eligibility criteria were allocated to three experimental cohorts: tDCS with joint mobilization (tDCS + JM); active joint mobilization (AJM); and tDCS with active joint mobilization (tDCS + AJM). Distribution across the treatment arms was accomplished through computerized randomization using specialized allocation software (Random allocation software program for Windows 2.0; Isfahan University, Isfahan, Iran). Participants were identified using a randomly generated two-digit code to maintain organizational integrity. The investigation employed a single-blind methodology, negating the temporal separation of intervention activities from intervention components conducted during non-overlapping periods. Assessment protocols were implemented at four discrete intervals (baseline, immediately post-intervention, completion of the treatment regimen, and follow-up) by an evaluator who maintained consistent involvement throughout the measurement phases.

### 2.5. Intervention

In this study, interventions were divided into three groups (tDCS + JM, AJM, tDCS + AJM). All the groups received identical treatment three times per week for four weeks, with each session lasting 15 min, for a total of 12 sessions.

#### 2.5.1. Transcranial Direct Current Stimulation Plus Joint Mobilization

**Figure 2:**
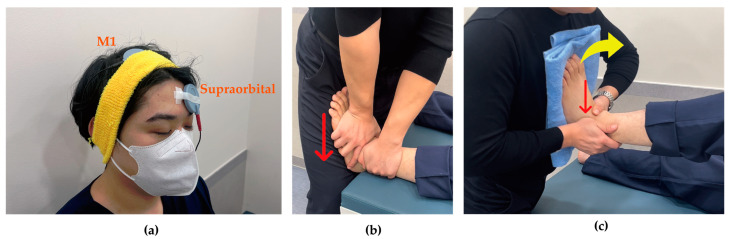
Intervention techniques. (**a**) Transcranial direct current stimulation: anode stimulates M1 contralateral to ankle instability and cathode attaches to supraorbital region ipsilateral to ankle instability; (**b**) Joint mobilization: as indicated by the red arrow, the talus applied a gliding force from superior to inferior with the tibia was fixed; (**c**) Active joint mobilization: as indicated by the red arrow, the talus and lateral malleolus applied anterior to posterior glide with dorsiflexion (yellow arrow).

tDCS was administered using The Brain Driver tDCS v2.1 (The Brain Driver Inc., Chicago, IL, USA). The equipment used in this study was identical to that employed in previous research, thus ensuring proven safety and reliability [27,28]. The electrode sponges were moistened with 0.9% physiological saline solution. The anodal electrode was attached to C3 or C4 according to the 10/20 international EEG system to stimulate primary motor cortex (M1) contralateral to the side of ankle instability, while the cathodal electrode was placed on the supraorbital region ipsilateral to the instability [29]. Stimulation was applied at an intensity of 2 mA for 15 min [24] (Figure 2a).

Joint mobilization was initiated 3 min after beginning tDCS and was performed at grade III according to Maitland’s classification, applying linear movement to the point of tissue resistance with high amplitude at the end range of joint motion and 1-s oscillations at mid-range. Participants assumed a supine position while the researcher grasped the talus of the affected ankle with one hand and the tibia with the other hand. The hand holding the talus was oriented toward the floor during the application of joint mobilization, which was administered for 10 min [30] (Figure 2b).

#### 2.5.2. Active Joint Mobilization

For active joint mobilization, participants were positioned in a supine position with the knee flexed on the side with instability. The researcher grasped the lateral malleolus with one hand and the talus with the other. The participant’s unstable foot was placed against the researcher’s sternum region while the researcher passively performed dorsiflexion, simultaneously sliding the talus and the lateral malleolus backward. After performing this passively 2–3 times, participants were instructed to actively perform dorsiflexion and plantarflexion according to the therapist’s direction. Once participants understood the movement pattern, they were verbally guided to perform active movements (Figure 2c).

#### 2.5.3. Transcranial Direct Current Stimulation Plus Active Joint Mobilization

Transcranial direct current stimulation with active joint mobilization involved the simultaneous application of tDCS and active joint mobilization. Following the same protocol as the tDCS with joint mobilization group, tDCS was first applied at an intensity of 2 mA for 3 min, after which active joint mobilization was initiated. The active joint mobilization was performed for 10 min, using the same technique described above.

### 2.6. Outcomes

For each group, outcome measures were assessed at multiple time points to evaluate the intervention efficacy. An immediate assessment was conducted following the first intervention session to determine acute effects. Post-intervention testing was performed after completing the 4-week intervention protocol. Additionally, a follow-up assessment was administered 4 weeks after the post-intervention testing to evaluate the durability of treatment effects (Figure 1).

#### 2.6.1. Dynamic Balance

Dynamic balance was assessed using the Y-Balance Test (YBT), a widely used evaluation tool for assessing dynamic balance ability in individuals with CAI. YBT offers a more efficient alternative to the Star Excursion Balance Test (SEBT), with excellent reliability (intraclass correlation coefficients ranging from 0.85 to 0.91) [31].

Leg length can influence the results when measuring dynamic balance using the YBT. Therefore, the reach distances in all three directions were normalized for analysis according to the established protocol [32]. Leg length was measured as the distance from the anterior superior iliac spine to the medial malleolus with the participant in a supine position. Participants were instructed to maintain their stance on the affected leg while reaching as far as possible with the contralateral leg in anterior, posteromedial, and posterolateral directions. Three practice trials were allowed, followed by three recorded trials, with the maximum reach distance used for analysis. The standardization formula is as follows:

$$Standardized\;ratio\;\left(\%\right)=\left[\frac{Sum\;of\;reach\;distanc\;in\;three\;dirctions}{Participaint^{\prime }s\;leg\;length\times 3}\right]\times 100$$

#### 2.6.2. Ankle Instability

Ankle instability was quantified using the CAIT, a validated instrument developed to assess functional ankle instability and its corresponding severity, which exhibits robust psychometric properties, including exceptional reliability and validity coefficients [33]. The comprehensive evaluation tool encompasses nine discrete parameters: nociceptive response, functional capacity during locomotion, rotational capability, ascending/descending graduated surfaces, unilateral stance maintenance, saltatory performance, stability on irregular terrain, talocrural joint inversion incidents, and proprioceptive recovery after inversion trauma. The assessment utilized a 30-point ordinal scale, with superior scores reflecting enhanced stability. According to the International Ankle Consortium, scores of <24 indicate clinically relevant ankle instability [34]. The established minimal clinically important difference threshold has been determined to be 3 points on the comprehensive 30-point evaluation metric [35].

#### 2.6.3. Range of Motion

The range of motion assessment focused on dorsiflexion range of motion (DFROM), a parameter commonly diminished in individuals with CAI. Quantification was performed using the weight-bearing lunge test methodology, employing an accelerometer sensor-based inclinometer integrated into a smartphone device (iPhone 13 mini, Apple Inc., Cupertino, CA, USA, 2021). Intra-rater reliability coefficients for digital inclinometer measurements have been established at 0.96–0.97, with minimal detectable change thresholds determined to be 3.7–3.8° [36]. Participants positioned the affected foot so that the calcaneus remained in contact with the supporting surface while the contralateral limb was positioned posteriorly at a standardized distance. During lunge execution, the second metatarsal was maintained parallel to the sagittal plane to ensure consistent calcaneal contact with the supporting surface. The affected limb was progressively advanced in 1 cm increments until patellar contact with the vertical surface was achieved, without eliciting a nociceptive response, at which point the smartphone inclinometer was placed along the tibial shaft for angular measurement [36].

#### 2.6.4. Static Balance

Static postural equilibrium was evaluated using a single-leg stance protocol to analyze the center of pressure (COP) displacement patterns via the APP-Coo-Test application accessible through the App Store platform on a mobile telecommunications device (iPhone 13 mini, Apple Inc., Cupertino, CA, USA, 2021). This application employs integrated gyroscopic sensor technology to quantify the trajectory of a digital indicator that represents positional shifts. The methodological implementation involved securing the telecommunication device to the sternum of the participant using an elastic circumferential restraint mechanism. Measurement acquisition occurred when the visual sensory input was maintained, while the limb contralateral to the affected extremity was elevated from the supporting surface. Within the application interface, parameters were configured to “feet together” within the static balance test module, with the assessment initiation command actuated simultaneously with contralateral limb elevation. Quantitative data extraction consisted of percentage values generated following a 10-s acquisition interval, with these metrics serving as representations of the participant’s center of pressure oscillation magnitude.

### 2.7. Data Analysis

Statistical analyses were performed using the SPSS software (version 21; IBM Corporation, Armonk, NY, USA, 2012). Homogeneity evaluation across participant cohorts was conducted using the chi-square test and one-way ANOVA, while participant demographic characteristics were elucidated using descriptive statistical parameters.

Normality of the scale variables was assessed through skewness and kurtosis, and all the values were within the acceptable range of ±2, supporting the assumption of normality for parametric testing. To investigate therapeutic efficacy, outcomes pertaining to dynamic balance, ankle instability, range of motion, and static balance were subjected to two-way repeated-measures ANOVA. This model included three components: the main effect of time (differences across multiple measurement points); main effect of group (differences among intervention groups); and time × group interaction effect (differential changes over time between groups). When significant interaction effects were identified, a univariate ANOVA was performed.

The effect magnitudes across measurement intervals between experimental conditions were quantified using paired-sample *t*-tests with the corresponding Cohen’s d calculations. Effect size interpretations adhered to conventional thresholds: values below 0.2 represent minimal effects; values between 0.2 and 0.5 indicate moderate effects; and values exceeding 0.8 denote substantial intervention effects. The alpha level for statistical significance was set at 0.05, and post-hoc analyses were conducted in accordance with Bonferroni correction protocols.

## 3. Results

**Figure 3:**
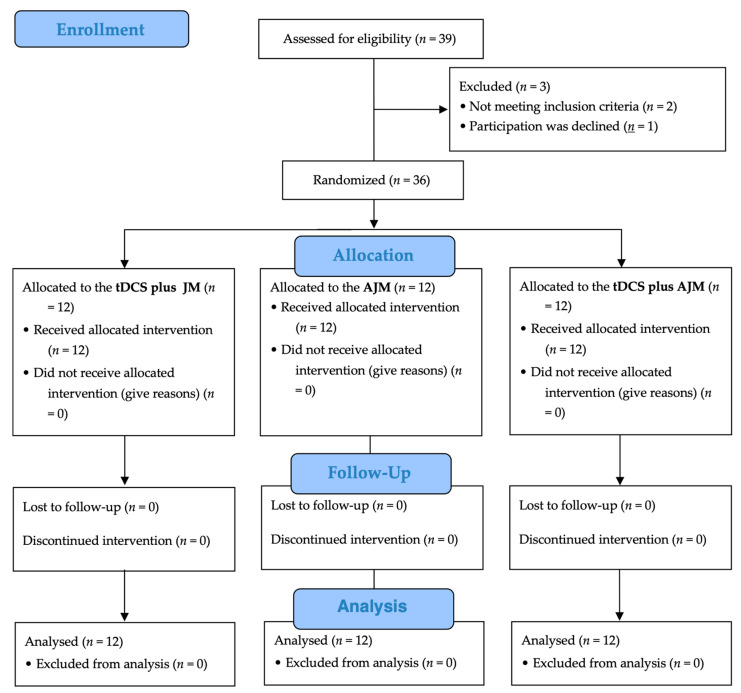
Flow diagram. AJM: active joint mobilization; JM: joint mobilization; tDCS: transcranial direct current stimulation.

Figure 3 shows a flow chart of this study based on the Consolidated Standards of Reporting Trials (CONSORT) guidelines. Of the 39 potential participants initially screened for eligibility, three were excluded (two did not meet the inclusion criteria and one declined participation). The remaining 36 participants were randomly assigned in equal numbers to one of three intervention groups: tDCS + JM (*n* = 12), AJM (*n* = 12), and tDCS + AJM (*n* = 12). All the participants received their allocated interventions, with no participants lost to follow-up or discontinuation of the intervention in any group. All the 36 enrolled participants (12 in each group) were included in the final analysis.

### 3.1. General Characteristics of the Participants

Table 1 presents the participants’ general characteristics. No statistically significant differences were observed among the three intervention groups regarding sex distribution, affected side, age demographics, leg length, or body mass index parameters (*p* > 0.05).

### 3.2. Dynamic Balance

In the intergroup comparison of dynamic balance across intervention methodologies, significant temporal variations were observed (*p* < 0.05); however, no significant time-by-group interaction effects were detected (*p* > 0.05).

When examining the effect sizes across measurement time points, the tDCS + AJM group exhibited the largest improvement in dynamic balance both immediately after the intervention (d = 0.929, 95% CI = 0.231, 1.598) and at the 4-week follow-up (d = 1.787, 95% CI = 0.843, 2.701) (Table 2).

### 3.3. Ankle Instability

Regarding the ankle instability parameters, intergroup comparisons revealed significant temporal variations (*p* < 0.05); however, no significant time-by-group interaction effects were observed (*p* > 0.05).

Effect size analysis across the measurement time points indicated that the tDCS + AJM group demonstrated superior improvement at both post-intervention (d = 2.230, 95% CI = 1.138, 3.296) and 4-week follow-up assessments (d = 1.772, 95% CI = 0.833, 2.681) (Table 2).

### 3.4. Range of Motion

For range of motion parameters, intergroup comparisons revealed significant temporal variations (*p* < 0.001); however, no significant time-by-group interaction effects were observed (*p* > 0.05).

The tDCS + AJM group exhibited the most pronounced gains in DFROM, both immediately post-intervention (d = 1.634, 95% CI = 0.739, 2.499) and at 4-week follow-up assessments (d = 3.300, 95% CI = 1.818, 4.761) (Table 2).

### 3.5. Static Balance

In the static balance assessment, intergroup comparisons revealed significant temporal variations (*p* < 0.001), although no significant time-by-group interaction effects were detected (*p* > 0.05).

Effect size analysis across measurement timepoints demonstrated that tDCS + AJM yielded the most substantial immediate post-intervention (d = 0.702, 95% CI = 0.054, 1.325) and at the 4-week follow-up assessments (d = 0.924, 95% CI = 0.228, 1.593) (Table 2).

## 4. Discussion

As studies highlighting improved balance capabilities resulting from cortical excitability regulation through non-invasive stimulation emerged, research investigating the effects of tDCS increased. This study aimed to examine the effects of tDCS combined with JM and AJM on adults with CAI. Assessments were conducted at baseline, immediate, post-intervention, and 4-week follow-up to determine whether the combined interventions would yield more effective outcomes.

To measure dynamic postural stability across interventions, we used the YBT, a streamlined version of the SEBT that measures dynamic balance using standardized values, accounting for the participants’ leg length. Dynamic balance showed significant time effects across all interventions; however, no significant time-by-group interaction effects were observed. When comparing effect sizes across measurement periods, the tDCS + AJM group exhibited the largest effect size in both the immediate and post-intervention assessments. Furthermore, large effect sizes were observed in all groups after intervention; only the tDCS groups demonstrated large effects immediately after intervention. Comparing the effect sizes between the tDCS + JM and AJM groups, similar effect sizes were observed after intervention, but the tDCS + JM group showed larger effect sizes at the immediate assessment. Significant differences (*p* < 0.05) in toe pinch strength, a critical component of lower limb motor function, were observed after applying tDCS combined with non-tDCS interventions, which appeared to influence dynamic balance associated with lower limb motor function [37]. Significant effects (*p* < 0.05) in YBT performance were reported in both the dominant and non-dominant feet in healthy young adults after applying tDCS [24]. These findings align with our results, supporting the notion that tDCS regulates the center-of-mass movement within the ankle support surface, a factor involved in dynamic postural stability.

Reflecting on our primary variable of dynamic balance, and considering our research objectives, meaningful differences were observed when comparing cortical activation through volitional movement versus top-down regulation through tDCS. Notably, the tDCS + JM group showed larger effect sizes than the AJM group in the immediate assessment, suggesting that interventions utilizing top-down regulatory mechanisms may have been more effective for immediate motor control enhancement.

We used the CAIT to assess changes in perceived ankle instability. Significant temporal improvements were observed across all time points; however, no significant time-by-group interactions were observed. The tDCS + AJM group demonstrated the largest effect size post-intervention and maintained this superiority at the 4-week follow-up assessment. When comparing the tDCS + JM and AJM groups, similar effect sizes were observed at both post-intervention and follow-up assessments.

The finding that the tDCS + AJM group showed larger effect sizes than the AJM group at follow-up suggested that tDCS application helped maintain intervention effects. Furthermore, considering that the MCID for CAIT is 3 points, the fact that all groups showed improvements exceeding 6 points post-intervention indicated that all intervention methods could contribute to functional recovery in patients with CAI, despite the absence of between-group differences. However, as the CAIT is a self-reported measure, it may be subject to external influences beyond the studied independent variables [20]. This suggested that the lack of between-group differences in CAIT results might have been influenced by external factors beyond the independent variables.

Ankle DFROM significantly improved over time across all intervention groups. When comparing effect sizes, tDCS + AJM group demonstrated the largest effect size in dorsiflexion range in both the immediate and post-intervention assessment.

Moderate effect sizes on DFROM have been reported when JM was applied without volitional movement in patients with ankle instability [38]. The higher effect sizes observed in groups receiving tDCS might be explained by the finding that applying tDCS reduced ankle pain perception in patients with ankle instability, thereby increasing the joint angle at which pain occurred during dorsiflexion and consequently increasing DFROM [39].

No significant differences (*p* > 0.05) in dorsiflexion angle were found after applying JM with volitional movement, with post-measurements conducted within 24–48 h [40]. The authors suggested that the effects of JM with volitional movements were short-lived. In contrast, our study measured the immediate effects directly after the intervention, which might explain our differing results regarding dorsiflexion changes. Notably, increased dorsiflexion angle enhances dynamic balance by expanding the range of movement of the body’s center of gravity [41]. This indicates that ankle DFROM is ultimately related to dynamic balance, our primary dependent variable.

To measure postural stability across interventions, we assessed static balance using an application capable of tracking COP movement during single-leg stance on the unstable foot. While static balance showed significant improvements over time, no time-by-group interaction was identified. Notably, the tDCS + AJM group demonstrated the largest effect sizes in static balance at both immediate and post-intervention assessments, indicating greater responsiveness to the combined intervention.

These results aligned with previous research showing that tDCS alone has no effect on static balance in young adults [42]. Although numerous studies have investigated the static balance deficits in patients with CAI, few have demonstrated improvements in static balance. The extent of postural control deficits has not been consistently measured when measuring postural control capabilities with eyes open in individuals with CAI [43]. Similarly, our study measured static balance with eyes open, which may have caused participants to rely more on visual information than on somatosensory input, potentially explaining our inconsistent results.

This study contributes significantly to the understanding of CAI rehabilitation by introducing a combined intervention approach that extends beyond traditional single-intervention methods. We assessed the effects of combined interventions across multiple dimensions, including dynamic balance, ankle instability, DFROM, and static balance, providing a more comprehensive analysis than previous studies that focused on individual interventions. Notable findings included the tDCS + AJM group showing the largest effect sizes for dynamic balance at both immediate and post-intervention assessments, sustained effects on ankle instability at 4-week follow-up, and superior outcomes in DFROM. Our combined intervention approach addressed limitations identified in previous research, such as moderate effect sizes with JM alone [38] and limited duration of effects with volitional movement-assisted JM [40]. The synergistic effects observed when combining findings on pain perception reduction through tDCS [39] with observations on enhanced dynamic balance through increased ankle ROM [41] suggest that integrating neurophysiological and functional approaches may be more effective for CAI rehabilitation than applying individual interventions.

A particularly noteworthy finding was the synergistic effect of the simultaneous application of JM and tDCS. The superior effect sizes of the tDCS + AJM group at both immediate and post-intervention assessments suggest its potential to overcome the limited duration associated with individual interventions. This appears to reflect the synergistic effects of cortical activation and proprioceptive sensory input on neuromuscular control enhancement.

However, this study has some limitations. Follow-up assessments to evaluate intervention durability were conducted only with CAIT, without reassessing ankle DFROM and balance parameters. Additionally, static balance was evaluated with participants’ eyes open, potentially causing participants to rely less on somatosensory input and more on visual information, possibly limiting the manifestation of neuromuscular control benefits of tDCS and volitional movement. Furthermore, the total sample size may have been insufficient to detect between-group differences, particularly given the number of groups and measurement time points. In addition, the follow-up period was relatively short compared to the typical duration in chronic rehabilitation trials, which may not fully capture the long-term retention of the intervention effects. Future studies measuring static balance in patients with CAI should consider blocking visual input to obtain more accurate results regarding static balance capabilities.

## 5. Conclusions

This study demonstrated that combining tDCS with active joint mobilization showed promising results for ankle instability rehabilitation. While all the intervention groups showed significant improvements in dynamic balance, ankle instability, range of motion, and static balance, the tDCS + AJM group consistently demonstrated larger effect sizes across most outcome measures. These findings suggest that integrating tDCS with traditional joint mobilization techniques can provide enhanced therapeutic benefits by simultaneously targeting cortical excitability and peripheral joint mechanics. Future research should address the limitations of this study by implementing more comprehensive follow-up assessments and refining testing protocols, particularly for static balance evaluation.

## Figures and Tables

**Table 1:** General characteristics of the participants.

Variables	tDCS + JM	AJM	tDCS + AJM	X^2^/t
Sex (*n*, %)				2.250
Male	2(16.7%)	6(50.0%)	5(41.7%)	
Female	10(83.3%)	6(50.0%)	7(58.3%)	
Affected side (*n*, %)				2.109
Left	7(58.3%)	5(41.7%)	9(75.0%)	
Right	5(41.7%)	7(58.3%)	3(25.0%)	
Age (years)	20.66 ± 1.72	20.91 ± 1.72	20.33 ± 1.23	0.412
BMI (kg/m^2^)	22.40 ± 5.06	21.39 ± 2.79	20.72 ± 2.98	0.611
Leg length (cm)	96.00 ± 4.36	97.36 ± 5.14	97.16 ± 4.87	0.277

Values are presented as mean ± standard deviation or number (%). AJM, active joint mobilization; BMI, body mass index; JM, joint mobilization; tDCS, transcranial direct current stimulation.

**Table 2:** Differences between groups by measurement time point for dynamic balance, CAIT, ROM, static balance.

Variables		tDCS + JM	AJM	tDCS + AJM	Time	Group	Time × Group
					F(*p*)	F(*p*)	F(*p*)
Dynamic balance	Baselines (A)	68.32 ± 8.50	66.09 ± 8.67	66.32 ± 4.98	55.818 (0.000)	0.238 (0.790)	0.507 (0.730)
	Immediate change (B)	73.57 ± 8.70	70.73 ± 5.58	72.41 ± 7.44			
	B-A ^a^	0.851 (0.171, 1.503)	0.587 (−0.390, 1.192)	0.929 (0.231, 1.598)			
	Post-test(C)	77.97 ± 8.94	79.79 ± 8.78	81.89 ± 8.64			
	C-A ^a^	1.484 (0.663, 2.278)	1.271 (0.484, 2.207)	1.787 (0.843, 2.701)			
Ankle instability	Baselines (A)	16.91 ± 4.07	16.58 ± 2.64	17.50 ± 2.96	84.851 (0.000)	0.566 (0.573)	0.241 (0.914)
	Post-test (B)	23.58 ± 4.44	24.16 ± 3.06	24.75 ± 3.69			
	B-A ^a^	1.585 (0.705, 2.434)	1.634 (0.739, 2.499)	2.230 (1.138, 3.296)			
	Follow-up (C)	22.50 ± 3.65	23.08 ± 2.42	24.33 ± 4.35			
	C-A ^a^	1.425 (0.594, 2.225)	1.412 (0.585, 2.209]	1.772 (0.833, 2.681)			
Range of Motion	Baselines (A)	20.16 ± 6.64	21.16 ± 10.32	25.08 ± 8.29	81.934 (0.000)	3.434 (0.440)	0.636 (0.639)
	Immediate change (B)	24.91 ± 10.12	26.50 ± 8.83	32.66 ± 9.12			
	B-A ^a^	0.754 (0.095, 1.387)	0.623 (−0.010, 1.233)	1.634 [0.739, 2.499]			
	Post-test(C)	32.66 ± 6.18	36.25 ± 6.04	41.75 ± 5.10			
	C-A ^a^	2.660 (1.415, 3.881)	1.594 (0.712, 2.446)	3.300 (1.818, 4.761)			
Static balance	Baselines (A)	77.42 ± 11.60	71.56 ± 12.51	78.50 ± 7.41	10.551 (0.000)	1.580 (0.221)	0.418 (0.795)
	Immediate change (B)	79.17 ± 8.53	75.41 ± 16.98	82.38 ± 6.01			
	B-A ^a^	0.261 (−0.320, 0.832)	0.365 (−0.228, 0.943)	0.702 (0.054, 1.325)			
	Post-test (C)	81.62 ± 11.92	79.67 ± 13.74	87.04 ± 5.81			
	C-A ^a^	0.387 (−0.209, 0.967)	0.866 (0.183, 1.521)	0.924 (0.228, 1.593)			

^a^ Cohen’s d and 95% confidence interval (lower limit, upper limit) for the pre-post difference. Values are presented as mean ± standard deviation. AJM, active joint mobilization; JM, joint mobilization; tDCS, transcranial direct current stimulation.

## Data Availability

The data presented in this study are available upon request from the corresponding author due to privacy reasons.

## Abbreviations

The following abbreviations are used in this manuscript:

AJM	Active joint mobilization
CAI	Chronic ankle instability
CAIT	Cumberland ankle instability tool
COP	Center of pressure
DFROM	Dorsiflexion range of motion
FAI	Functional ankle instability
JM	Joint mobilization
MAI	Mechanical ankle instability
MCID	Minimal clinically important difference
NIBS	Non-invasive brain stimulation
SEBT	Star excursion balance test
tDCS	Transcranial direct current stimulation
YBT	Y-balance test
